# Rate, Mode, Reasons and Factors Associated With Re‐Presentation in People Diagnosed With Musculoskeletal Conditions at a Single Emergency Department: A Cross‐Sectional Exploratory Study

**DOI:** 10.1111/1742-6723.70128

**Published:** 2025-08-29

**Authors:** Patricia Slapp, Linda Spencer, Rob Waller, Karen Richards, Anne Smith, Nic Saraceni, Piers Truter

**Affiliations:** ^1^ Curtin School of Allied Health Curtin University Bentley Australia; ^2^ Physiotherapy Department Rockingham General Hospital Rockingham Australia; ^3^ Kaartdijin Innovation Centre South Metropolitan Health Service Murdoch Australia; ^4^ School of Health Sciences University of Notre Dame Australia Fremantle Australia

**Keywords:** emergency service, fracture, hospital, musculoskeletal pain, physiotherapy, re‐presentation

## Abstract

**Objective:**

To explore the rate, mode, and reasons for re‐presentations for emergency department (ED) patients with musculoskeletal diagnoses and examine factors associated with increased odds of re‐presentation.

**Methods:**

A retrospective cross‐sectional audit of re‐presentation patterns for patients with musculoskeletal diagnoses presenting to the study ED in 2023.

The study ED was in a secondary hospital in Perth, Western Australia, which operates a diversion pathway (daily, 10 am–6 pm) for patients with musculoskeletal diagnoses.

**Results:**

In 2023, 3677 patients with musculoskeletal diagnoses were diverted from the ED, 972 provided research consent and of those, 143 (14.7%) re‐presented. Importantly, 10 (1.0%) patients re‐presented to the ED itself, and 133 (13.7%) re‐presented to the physiotherapy outpatient diversion clinic.

There were 65 scheduled and 78 unscheduled re‐presentations, with telehealth the preferred mode of contact (*n* = 86, 60.1%). Clinician diagnostic uncertainty, identified patient psychosocial issues, and concern that the patient would re‐present to the ED most commonly resulted in scheduled re‐presentation (*n* = 31, 47.7%). Unscheduled re‐presentations focused on administrative inquiries (e.g., hospital referrals, medical certificates) (*n* = 31, 39.7%) and concern for symptoms (*n* = 25, 32.1%).

Older age, high pain severity, and lower limb affected body region were associated with increased odds of re‐presentation.

**Conclusions:**

ED clinicians may reduce re‐presentation by employing targeted strategies such as shared decision‐making about pain management, ensuring a shared understanding of the diagnosis, or stage of the diagnostic process and likely course of symptoms. Further, a follow‐up plan that is clinically indicated and patient acceptable may be critical for those with increased odds of re‐representation.

## 1 Introduction

In Australia in 2023/24 there were 9.0 million presentations to public hospital emergency departments (ED) [1]. Many people make multiple presentations to the ED, increasing the burden on already overcrowded health services. Dinh et al. [2] identified that 48.9% of people presenting to the ED had a prior presentation in the previous 12 months for one or more health conditions. This probably reflects frequent use of the ED rather than re‐presentation, which is a repeat visit for the same health complaint. ED re‐presentation rates within 72 h are estimated at 2.7%–4.9% [2, 3, 4]. Patients with musculoskeletal (MSK) diagnoses make up 8.7%–13.8% of all ED presentations [5, 6] and people with injury and MSK diagnoses are the most common complaints returning the to ED, accounting for 28.1% of all re‐presentations within 72 h [2].

There are many factors associated with ED re‐presentation. The most common factors are illness‐related (49%) [4] and linked to complications, disease progression and a need for additional diagnostics. The second factors (41%) and the most challenging to influence are patient‐related and include non‐compliance with prescribed care, failure to access primary care follow‐up, leaving without being seen, poor mental health and worry about health [3, 4]. Clinician factors account for 10% of re‐presentations and include treatment error, misdiagnosis and poor pain control [3, 4]. Further, clinicians correctly communicating prognosis, providing correct treatment and agreeing with the patient on suitable follow up may have critical associations with re‐presentation rates [7]. Aside from these factors, some conditions (e.g., abdominal pain) are independently associated with re‐presentation.

While there is a developing general picture of why people re‐present to the ED, there are no studies that specifically consider the situation and needs of patients with MSK diagnoses diverted from ED. Understanding the drivers for re‐presentation in this population will help develop targeted strategies to reduce ED burden.

The aims of this study conducted at a Western Australian hospital ED were:

Aim 1. To describe the rate, mode, reasons, and outcomes for both scheduled and unscheduled re‐presentations for patients with MSK diagnoses presenting to and diverted from the study ED.

Aim 2. To investigate factors associated with re‐presentation for patients with MSK diagnoses presenting to, and diverted from, the study ED.

## 2 Methods

### 2.1 Study Design

This was a retrospective cross‐sectional audit of re‐presentation patterns for patients with an MSK diagnosis presenting to and diverted from the study ED.

### 2.2 Setting

The study was conducted at the Rockingham General Hospital ED, a 229‐bed hospital in Perth, Western Australia that had 61,980 total ED presentations in 2023–24 [1]. This ED operates a diversion pathway (daily, 10 am–6 pm) for patients with MSK diagnoses [8, 9]. The diversion pathway, led by Advanced Scope Physiotherapists (ASP), allows suitable patients to leave the ED and transit to an external physiotherapy outpatient clinic for assessment and management. The guiding diversion eligibility criteria are people with an MSK presenting complaint, aged 8–65, and triaged to Australasian Triage Categories (ATS) 3–5 [8] (Box 1).

BOX 1 **Procedure: Emergency Department Musculoskeletal Diversion Pathway.**

A triaging advanced scope physiotherapist (ASP) was stationed in the ED waiting room and the treating team (a second ASP and senior physiotherapist) was in the hospital physiotherapy outpatient clinic. All patients went through normal ED triage procedures and a demographic check with ED clerks. Patients who met eligibility criteria for diversion were identified from the presenting complaint information entered into the Emergency Department Information System (EDIS). The ASP approached the patient in the ED waiting room, took a brief history, undertook a triage assessment, and, if deemed eligible, offered diversion to the ASP‐managed outpatient clinic. There was a two‐part consent process for the diversion pathway and to participate in the research project. Once patient consent for diversion was obtained, they were registered in a Research Electronic Data Capture (REDCap) database, which also provided a digital clinical workflow and medical record authoring tool for diversion pathway clinicians. Patients were given the option to decline diversion and receive usual ED care, remaining in the waiting room to follow usual department procedures. Analgesia was provided to all patients in the waiting room as per usual ED protocol. Patients consenting for diversion were discharged from the ED and admitted to the diversion pathway. Indicated imaging (e.g., radiographs of the limbs) was ordered by the triaging ASP and the indicated images were taken as patients transited to the outpatient clinic. Upon arrival, patient care was transferred to the treating ASP or senior physiotherapist, depending on the clinical complexity of the patient's presentation. The diversion pathway team accessed a digital clinical handover, assessed the patient, reviewed imaging, provided a diagnosis and initiated appropriate evidence‐based care. The physiotherapist was able to consult with the ED medical team or hospital specialty services (e.g., Orthopaedics). Following care, patients were discharged home with a management plan and safety net contact details for the physiotherapy outpatient clinic.

Excerpt from [8].

Following management, the diversion pathway clinicians provide patients with safety net options that facilitate unscheduled (patient‐initiated) and scheduled (clinician‐initiated) re‐presentations. Guidance provided to every patient is to directly contact the outpatient clinic by telephone, email, or in person, in preference to returning to the ED, if they need further support related to their diagnosis. In addition, if indicated, diversion pathway clinicians can offer in‐person or telehealth follow‐up outpatient appointments to patients. The diversion pathway clinicians manage scheduled and unscheduled re‐presentations as part of their daily outpatient clinic operations. The diversion pathway clinicians also review all radiology once reported and schedule re‐presentation if the formal report differs from the initial presentation radiology interpretation by the treating clinician. Patients who were diverted can choose to re‐present to the main ED.

### 2.3 Participants

For this study, patients diverted between 1 January 2023 and 31 December 2023 were included if they provided informed consent to allow their data to be used for research purposes. Included patients were categorised into three subgroups based on whether they re‐presented within 28 days following their initial visit:

- Scheduled (clinician‐initiated): patient had an in‐person or telehealth physiotherapy outpatient clinic appointment, as planned during the initial presentation.
- Unscheduled (patient (or guardian) initiated): patient re‐presented to ED or activated the safety net process, leading to an unplanned in‐person or telehealth physiotherapy outpatient clinic appointment (telehealth/in‐person).
- Did not re‐present: patient did not re‐present to the ED or physiotherapy outpatient clinic.

### 2.4 Outcome Assessment

#### 2.4.1 Data Collection Procedures

Data was drawn from a Research Electronic Data Capture (REDCap) database hosted at Western Australia Department of Health. This software was used by diversion pathway clinicians to facilitate clinical workflows, document clinical notes, and administer a patient satisfaction questionnaire [10] following patient discharge. Patients who consented to participate in research were also sent a questionnaire that included the EQ‐5D (with age appropriate and guardian completion versions) [11] and a single item literacy screener question [12]. Detailed information is available in Appendix B.

### 2.5 Outcomes

#### 2.5.1 Aim 1 Describing Re‐Presentation

Rate of re‐presentation was defined by the number of patients who re‐presented as a proportion of all patients included in the study, over 1 year. All diverted participants were audited against the ED information system (EDIS) and hospital administrative systems (WebPAS) to ensure all re‐presentations to the ED and diversion pathway were recorded.

Mode of re‐presentation was a categorisation into one of three re‐representation types:

- in person re‐presentation to the hospital ED
- in‐person re‐presentation to the outpatient clinic
- telehealth re‐representation to the outpatient clinic (either telephone call or email request)

Reasons for re‐presentation and subsequent outcomes were developed through an inductive process. Two researchers (LS and PS) independently reviewed the clinical notes for every patient that re‐presented and categorised reasons for re‐presentation. The authors were blinded to each other's results. A list of primary and secondary reasons for scheduled and unscheduled re‐presentation was then inductively developed and agreed upon by consensus with the research team (refer to Appendix A).

#### 2.5.2 Aim 2 Regression Analysis

As this study was an exploratory investigation of associations between patient characteristics and re‐presentation, some variables selected for regression modelling were based on previously reported re‐presentation associations [2, 3, 13, 14]. Additional variables were selected based on the consensus of expert MSK clinicians working clinically in the diversion pathway.

Data for all patients were obtained from clinical notes via REDCap and included *age, sex at birth, area injured, current smoking status, medications*, type of *MSK diagnosis* (traumatic, atraumatic), plaster immobilisation, imaging, postcode, occupation, *adverse events*, and *outcome of care*. Adverse events were defined as an injury caused by medical management rather than by the underlying disease or condition of the patient [15]. ATS category for the index ED presentation was extracted from EDIS.

Four regression analysis variables were drawn from items in online questionnaires completed by participants at their initial visit. These were: pain/discomfort (EQ‐5D), anxiety/depression (EQ‐5D) [11], health literacy (Literacy screener) [12], and patient satisfaction (SAPS) [10]. Please see Appendix B for a detailed description of regression analysis variables.

### 2.6 Data Analysis

#### 2.6.1 Aim 1 Describing Re‐Presentation

All statistical analysis was performed using IBM SPSS (version 29.0).

The rate of re‐presentation is reported as a proportion of those who re‐presented compared to all patients included in the study, over 1 year.

The mode of re‐presentation is reported as a proportion of those who re‐presented through a specific mode, compared to the number of patients who re‐represented.

The reasons for re‐presentation are categorised into scheduled and unscheduled representations.

1. Reasons for scheduled re‐presentation are reported as proportions of the total number of scheduled re‐presentations.
2. Reasons for unscheduled re‐presentation are reported as proportions of the total number of unscheduled re‐presentations.

Patients who experienced a change in management are reported as a proportion of the total number of patients who re‐presented. It is further sub‐categorised by scheduled and unscheduled re‐presentations and reported as a proportion.

As this study was exploratory, analyses were conducted using the available data without imputation. Where data was missing, the number analysed were reported.

#### 2.6.2 Aim 2 Regression Analysis

Re‐presentation was considered as a binary ‘Did’/‘Did not’ variable. Binary logistic regression analysis (adjusted for age and sex) was used to estimate associations between independent variables (patient characteristics) and re‐presentation (dependent variable), and odds ratios presented with accompanying 95% confidence intervals and accompanying *p*‐values. Age and sex were identified as potential confounders due to their association with the outcome (re‐presentation). Therefore, subsequent logistic regression models adjusted for age and sex and both adjusted and unadjusted odds ratios for patient characteristics were reported. A final multivariable logistic regression model was estimated which included those patient characteristics for which there was at least weak statistical evidence (at *p* < 0.10) for an association with re‐presentation after adjustment for age and sex. For Aim 2, assuming a re‐presentation probability of 0.15, a sample size of 900 participants enables detection of odds ratios of 1.3 or greater with 80% power and *α* = 0.05.

## 3 Ethics

Ethics approval for this study was obtained from South Metropolitan Area Health Service Human Research Ethics Committee (HREC) (RGS5279) and Curtin University Human Research Ethics Committee (HRE2022‐0561).

## 4 Results

### 4.1 Aim 1

**FIGURE 1:**
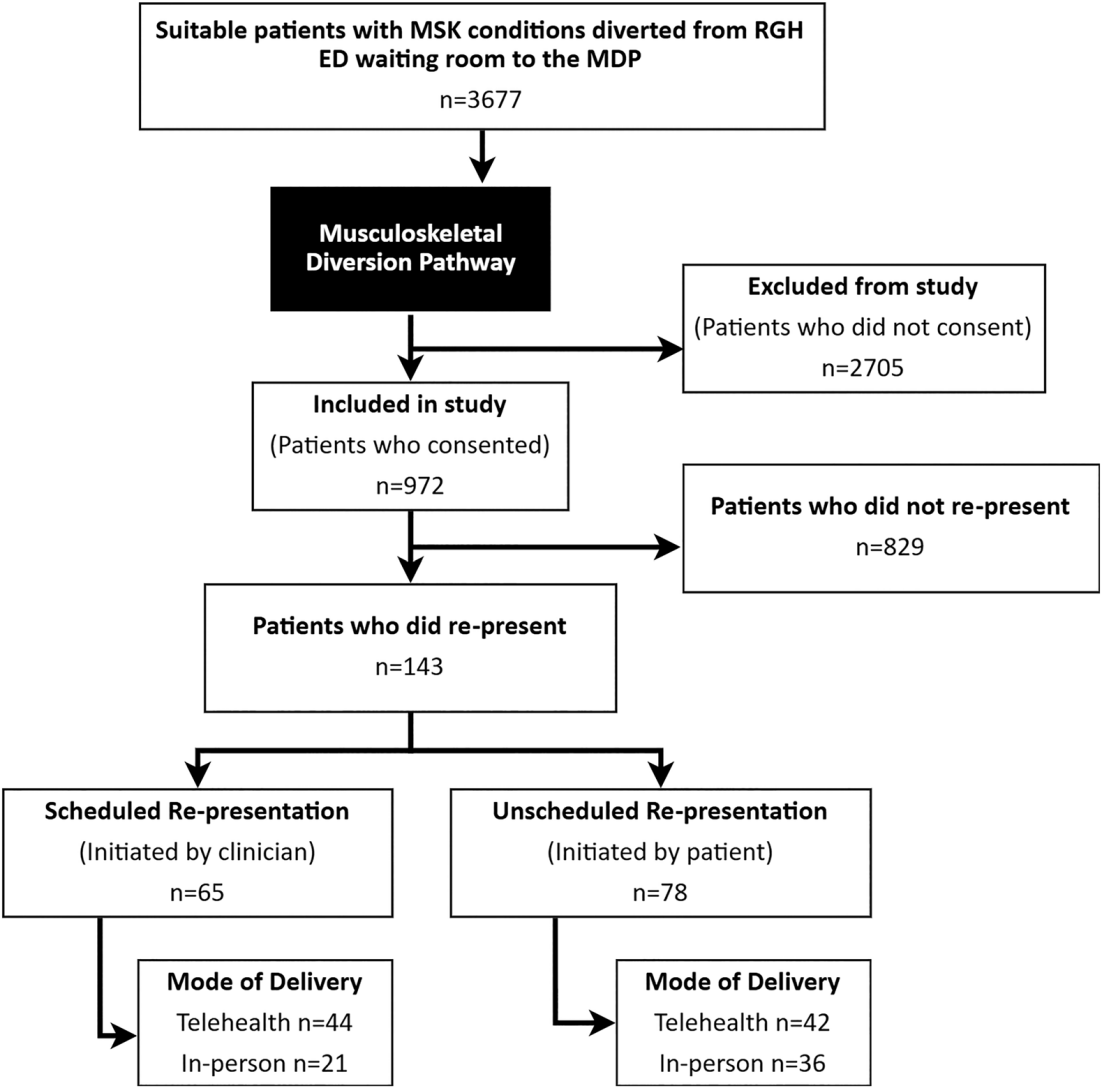
Flowchart showing included participants and re‐presentation sub‐groups.

In the study period, 3677 patients with MSK diagnoses were diverted from the study ED waiting room. The diversion pathway is permanently established in the study hospital, known to local residents, and data collection is integrated into diverted patient care. The rate of patients declining diversion is not measured because it is unusual for people to decline diversion and does not fit into patient care processes. Of the diverted patients, 972 provided research consent, and of these, 143 (14.7%) re‐presented (Figure 1).

Importantly, 10 (1.0%) patients re‐presented to the ED itself, and 133 (13.7%) re‐presented to the outpatient clinic. The number of re‐presentations was once (107%–11%), twice (27%–2.8%), and three or more times (9%–0.9%). There were no recorded adverse events. However, one patient who re‐presented went on to have further investigations and was subsequently diagnosed with deep vein thrombosis and pulmonary embolus. Telehealth (60.1%) was the preferred mode of re‐presentation for both scheduled and unscheduled re‐presentations. Patient characteristics between the scheduled and unscheduled re‐presenters differed only in the rate of plaster immobilisation (scheduled 1.5% vs. unscheduled 19.0%) and patient satisfaction (scheduled 91.6% vs. unscheduled 100%) (see Appendix C, Table C1).

The reasons for re‐presentation are shown in Table 1.

**TABLE 1:** Primary reasons for scheduled and unscheduled re‐presentation and management outcome.

		Did re‐presentation result in a change in management?	
		Change	Nil Change
Reasons for re‐presentation	n (%)	*n*Total = 31	Total *n* = 112
**Scheduled re‐presentation**.	**Total *n* = 65**	*n* = 25 (80.6)	*n* = 40 (35.7)
To confirm patient progress or provide additional support	31 (47.7)	7	24
Further investigation/diagnostics	16 (24.6)	4	12
Radiology results	13 (20)	11	2
Referral action required	5 (7.7)	3	2
**Unscheduled re‐presentation**	**Total *n* = 78**	*n* = 6 (19.4)	*n* = 72 (64.3)
Follow up/administrative	31 (39.7)	0	31
Concern for symptoms	25 (32.1)	4	21
Splinting review	16 (20.5)	2	14
Additional equipment needs	6 (7.7)	0	6

#### 4.1.1 Scheduled Re‐Presentation (Clinician Initiated)

Clinician uncertainty about patient progress or planned additional support for the patient was the most common reason for scheduled re‐presentation (*n* = 31/65, 47.7%). The offer of extra care was motivated by psychosocial concerns or where the patient was deemed at risk of representation to the ED.

#### 4.1.2 Unscheduled Re‐Presentation (Patient Initiated)

Patients mostly initiated re‐presentation via the safety net process (email *n* = 37, 47.4% and telephone *n* = 25, 32.1%) compared to re‐presenting in person (*n* = 16, 20.5%). Ten patients re‐presented in person to the Hospital ED. Reasons for unscheduled re‐presentation were mainly follow‐up/administrative enquiries (*n* = 31, 39.7%) or concern for symptoms (*n* = 25, 32.1%). Of these 31 follow‐up/administrative enquiries, 10 (32.3%) related to questions about hospital specialty referrals and 11 (35.5%) were regarding medical certificates.

#### 4.1.3 Change in Management

Change in management occurred in 31 of the 143 (21.7%) re‐presentations (see Table 1). This was more common following scheduled re‐presentations (*n* = 25/33, 80.6%) than unscheduled (*n* = 6/33, 19.4%). In scheduled re‐presentation, changes in management were mostly related to review after radiology was formally reported (*n* = 11) followed by additional support deemed necessary (e.g., GP follow‐up for pain management) (*n* = 7). In unscheduled re‐presentation, changes in management were mostly related to concern for symptoms (*n* = 4) followed by splinting review (*n* = 2).

### 4.2 Aim 2

When adjusted for sex, increasing age was associated with re‐presentation, such that the odds of re‐presentation increased by 1% for each year of age (OR 1.01, 95% CI 1.00–1.02, Table 2). When adjusted for age and sex, high pain severity at initial presentation was also associated with re‐presentation compared to patients with low pain severity (OR 2.16, 95% CI: 1.26–3.17). Having a lower limb affected compared to an upper limb affected was associated with increased odds (OR 1.54, 95% CI: 1.08–2.32) of re‐presentation.

**TABLE 2:** Characteristics of people presenting to ED who were diverted to the Musculoskeletal Diversion Pathway and the unadjusted and adjusted (age and sex) univariable associations between patient characteristics and the odds of re‐presentation.

Variable	Total	Did not re‐present	Did re‐present	Unadjusted	Adjusted (age, sex)	
	*n* = 972	*n* = 829	*n* = 143	OR (95% CI)	OR (95% CI)	*p* ^b^
Age^a^	22.7 (13–46)	21.6 (13–44)	31.3 (15–51)	1.01 (1.00–1.02)	**1.01 (1.00–1.02)**	**0.017** *
Sex at birth, female	491 (50.5)	407 (49.1)	84 (58.7)	1.47 (1.03–2.11)	1.38 (0.96 to 2.0)	0.077
Body area affected						0.059
Upper limb	488 (50.2)	401 (48.4)	87 (60.8)	Reference	Reference	
Lower limb	451 (46.4)	401 (48.4)	50 (35)	1.74 (1.19–2.53) 0.96	1.58 (1.08–2.32)	
Spinal	33 (3.4)	27 (3.2)	6 (4.2)	1.78 (0.70–4.53)	1.54 (0.60–3.96)	
Current smoker, yes	85 (8.7)	73 (8.8)	12 (8.4)	0.95 (0.50–1.80)	0.81 (0.42–1.55)	0.522
Number of medications						
Nil	604 (62.1)	526 (63.4)	78 (54.5)	Reference	Reference	0.491
1–3	342 (35.2)	284 (34.2)	58 (40.6)	1.38 (0.95–1.20)	1.12 (0.74–1.70)	—
4+	26 (2.7)	19 (2.3)	7 (4.9)	2.48 (1.01–6.10)	1.76 (0.68–4.58)	—
Type of injury						
Atraumatic				Reference	Reference	—
Traumatic	912 (93.8)	782 (94.3)	130 (90.9)	0.60 (0.32–1.14)	0.72 (0.37–1.40)	0.333
Plaster immobilisation, yes	130 (13.4)	110 (13.3)	20 (14)	1.06 (0.64–1.78)	1.16 (0.68–1.94)	0.585
Received imaging, yes	775 (79.7)	667 (80.5)	108 (75.5)	0.75 (0.49–1.14)	0.84 (0.54–1.29)	0.417
Triage Score, ATS						
Higher acuity [1, 2]				Reference	Reference	—
Lower acuity [3, 4]	841 (86.5)	720 (87)	121 (85)	0.83 (0.51–1.37)	0.82 (0.50–1.35)	0.445
Index of relative socioeconomic disadvantage (IRSD)						
More disadvantage (IRSD 1–5)				Reference	Reference	—
Less disadvantage (IRSD 6–10)	402 (41.4)	344 (41.5)	58 (40.6)	0.96 (0.67–1.38)	0.96 (0.67–1.39)	0.832
Occupation (Grouped)						
Other/unemployed/pensioner	97 (10)	83 (10)	14 (10)	Reference	Reference	0.362
Student	424 (43.6)	369 (44.5)	55 (38.5)	0.88 (0.47–1.66)	2.27 (0.90–5.76)	—
Sedentary	100 (10.3)	84 (10.1)	16 (11.2)	1.13 (0.52–2.46)	1.35 (0.61–3.00)	—
Standing/physical/heavy	351 (36.1)	293 (35.3)	58 (40.6)	1.17 (0.62–2.21)	1.62 (0.83–3.19)	—
	*n* = 873	*n* = 743	*n* = 130			
EQ5D pain severity						
Low (No or slight)	263 (30.1)	235 (31.6)	28 (21.5)	Reference	Reference	**0.019** *
Moderate	432 (49.5)	367 (49.4)	65 (50.0)	1.49 (0.92–2.38) 3.11	1.46 (0.92–2.36)	—
High (severe or extreme)	178 (20.4)	141 (19.0)	37 (28.5)	2.20 (1.29–3.75)	**2.16 (1.26–3.71)**	—
	*n* = 872	*n* = 742	*n* = 130			
EQ‐5D anxiety/depression						
Moderate–extreme				Reference	Reference	—
No or slight	762 (87.4)	650 (87.6)	112 (86.2)	0.88 (0.51–1.52)	0.95 (0.55–1.64)	0.849
	*n* = 871	*n* = 741	*n* = 130			
Health literacy						
Low				Reference	Reference	—
High	774 (88.9)	653 (88.1)	121 (93.1)	1.81 (0.89–3.70)	1.67 (0.81–3.42)	0.162
	*n* = 625	*n* = 520	*n* = 105			
Satisfaction						
Satisfied, yes	611 (97.8)	510 (98)	101 (96)	0.49 (0.15–1.61)	0.50 (0.15–1.64)	0.254

*Note:* Presented as *n* (%) unless otherwise specified. The bold values indicate a significant level of evidence for association.

^a^ Median, interquartile range.

^b^ *p* for group difference, adjusted for age and sex.

* Statistically significant.

Table 3 presents a final fully adjusted model including age, sex at birth, pain severity and body area affected. Older age, high pain severity and lower limb affected body region were independently associated with re‐presentation.

**TABLE 3:** Multivariable associations between patient characteristics and the odds of re‐presentation.

Variable	Odds ratio (95% CI)	*p* *
Age (years)	1.01 (1.00–1.02)	**0.055**
Sex at birth, female	1.26 (0.86–1.86)	0.242
Pain severity^a^		
Low	Reference	—
Moderate	1.39 (0.86–2.24)	0.179
High	1.93 (1.11–3.35)	**0.019**
Body area affected		
Upper limb	Reference	—
Lower limb	1.54 (1.02–2.33)	**0.038**
Spine	1.80 (0.67–4.84)	0.242

*Note:* Low = no/slight pain, moderate = moderate pain, high = severe/extreme pain. The bold values indicate a significant level of evidence for association.

^a^ Based on collapsed 5‐point Likert item in EQ‐5D.

* Level of evidence for association.

When pain severity at initial presentation was high compared to low, patients had 93% higher odds for re‐presentation, and 54% higher odds of re‐presentation when the lower limb was affected compared to the upper limb.

## 5 Discussion

The ED diversion pathway offers a unique opportunity to investigate re‐representation in ED patients with MSK diagnoses. The 28‐day re‐presentation rate of 14.7% is higher than the reported rate of 4.9% [3] for all ED patients. However, only 1.0% re‐presented in person to the ED, with most preferring a telehealth or in‐person follow‐up in the physiotherapy outpatient clinic. The most common reason for scheduled and unscheduled re‐presentation was concern about diagnosis and/or severity of symptoms. For clinicians, this may highlight the challenges of definitive MSK diagnosis in ED. The higher rate of change in patient management in scheduled (*n* = 25/31, 80.6%) compared to unscheduled (*n* = 6/31, 19.4%) re‐presentations may suggest that follow‐up visits improve diagnostic ability and therefore definitive management plans can be provided. For patients, the concern about symptoms leading to unscheduled re‐presentation aligns with the association of older age, higher pain severity, and lower limb injuries, which reduce mobility, as independent risk factors that increased the odds of re‐presentation.

One advantage of diverting people from the ED to an outpatient clinic is the capacity for clinicians to offer follow‐up appointments. At the index presentation there are diagnostic challenges with many acute MSK conditions, due to pain, swelling and guarding behaviours. ED clinicians often make decisions with incomplete information and without knowing the symptom trajectory. A working diagnosis is often made, which can change on review when a complete examination is possible or with the availability of expert imaging review [16]. ED clinicians may ‘play it safe’ with acute MSK conditions on the index presentation, and refer patients to outpatient specialist orthopaedic review based on working diagnosis [17]. This creates unnecessary referrals [16] and contributes to outpatient clinic crowding [18]. As this study demonstrates, continuity of care is ideal to complete the diagnostic process and can clarify the need for referral to hospital services. Continuity of care is also ideal for patients with high levels of pain and distress, where a planned follow‐up could prevent an in‐person return to the ED [4].

From a patient's perspective, negotiating a comprehensive patient‐centered initial management plan, including planned follow‐up that the patient accepts as appropriate, may be important to prevent re‐representation to the ED [4, 7]. Patient concern about symptoms was a leading cause of re‐representation in this study, with a higher rate than previously reported [4]. The physiotherapy outpatient clinic is convenient for patients, who can initiate safety net processes by telephone call or email. This convenience is probably part of the reason for the higher rate of re‐presentation. In this study, patients with MSK diagnoses also had unmet information and administrative needs relating to their hospital care pathways (e.g., communication from and access to outpatient specialist services).

A novel finding of this study is that patients with MSK diagnoses who were of older age, high pain severity, or had sustained a lower limb injury were more likely to re‐present. Dinh et al. [2] found that 72 h ED re‐presentation rates were highest in 0–4‐year‐old patients, but there was no association with increasing age. Referral and attendance to outpatient physiotherapy is, however, associated with lower ED re‐presentation rates in older adults [19]. Considering pain, people with low back pain receiving an opioid in the ED is associated with increased odds of re‐presentation [14]. This may not describe the issue completely, as van der Linden et al. [4] found that a lack of scripted analgesia was linked to re‐presentation. Further research is needed to clarify if differences in ED analgesia and discharge scripting are linked to re‐representation. Nonetheless, there are clearly actions that clinicians can take on an index ED presentation to reduce the odds of representation, which align with MSK care guidelines [20]. These include focusing on communication, particularly shared decision‐making, taking time to explain the diagnosis and likely course of symptoms, and agreeing on a pain management plan that includes non‐pharmacological elements. Further, agreeing on a follow‐up plan that is clinically indicated and patient acceptable may be critical to prevent re‐presentation.

## 6 Limitations

This study considered patients experiencing care and the specific safety net processes for a novel ED MSK diversion pathway in a secondary hospital in Western Australia. The results may not be broadly generalisable to the typical or international ED settings. Re‐presentation was only considered in the context of the study hospital, and it is unknown if the patients presented to another ED or connected to primary care.

## 7 Conclusions

The needs of patients presenting with MSK diagnoses may not be met by traditional ED services. When easily accessible, nearly 15% of patients accessed additional care following their index visit, highlighting a possible unmet need. Higher pain severity, older age, and lower limb injuries were independently associated with significantly increasing the odds of requiring additional care.

## Ethics Statement

Ethics approval was obtained from the South Metropolitan Area Health Service (SMHS) Human Research Ethics Committee (RGS5279) and Curtin University Human Research Ethics Committee (HRE2022‐0561).

## Conflicts of Interest

The authors declare no conflicts of interest.

## Data Availability

The data that support the findings of this study are available on request from the corresponding author. The data are not publicly available due to privacy or ethical restrictions.

## Appendix A Aim 1: Reasons for Re‐Presentation

An independently categorised list of primary and secondary reasons for scheduled and unscheduled re‐presentation was inductively developed from clinical notes by (PS) and (LS). There were no discrepancies between categorised lists; therefore, a third person was not required to reach an agreement.

### Scheduled (Initiated by the Clinician)

1. **Concern and additional support**
  Within this category, secondary reasons included yellow flags or those at risk of a poor trajectory, prevention of re‐presentation directly to ED, checking progress, symptoms and recovery, and provision of pain management education.
2. **Further investigation and/or diagnostics**
  This category included patients that were unable to be fully assessed during their initial appointment, diagnostic refinement, and additional screening for further injuries or red flags (e.g., deep vein thrombosis)
3. **Radiology or X‐Ray results prompted re‐presentation**
  This category was related to any patients who required further imaging, or when the results from their radiology or X‐ray prompted the clinician to contact the patient. This included education about the imaging results and any associated changes in management.
4. **Referral action required (either additional or referral rejected)**
  This category relates to any patients who required additional referrals to specialist services, outpatient physiotherapy, GP, or if their referral had been rejected for any reason.

### Unscheduled (Initiated by the Patient)

1. **Follow up and/or administrative enquiries**
  This category spans all enquiries related to follow‐up questions regarding their condition (e.g., ability to drive, return to work or imaging query), requests for medical certificates or discharge summaries, and questions related to referral appointments with other services.
2. **Concern for symptoms**
  This category included worsening symptoms (including swelling), worsening or stable pain, and neurological symptoms.
3. **Splinting review**
  This category was related to patients requesting review for an ill‐fitting splint, or replacement of their plaster splint due to damage (wet plaster).
4. **Additional equipment needs**
  This category relates to any replacement of equipment (due to loss, fault or damage) or any additional equipment that may be required in relation to a change in management (e.g., cam boot or crutches).

## Appendix B Regression Analysis Variables

1. **Age (Continuous, IQR)**

Age was used as a continuous variable as odds of re‐presentation showed a linear relationship.

Increase with age when split into deciles. Data source: EDIS

2 **Sex (male, female)** Data source: EDIS
3 **Body Area Affected (upper limb, lower limb, spinal)**
  Categories were collapsed from a large number of specific diagnoses into three categories (upper limb, lower limb and spinal) based on modelling from a previous study [8]. Data source: REDCap database
4 **Current Smoker (yes, no)** Data source: REDCap database
5 **Current Medications (no meds, 1–3 meds, 4 or more)**
  Number of current medications has been used in prior study as an exposure variable. We considered the clinical utility of polypharmacy scoring and used number of medications as a proxy to gain a sense of possible co‐morbidities as we did not have complete information regarding comorbidities within the dataset. Data source: REDCap database
6 **Traumatic Injury (traumatic, atraumatic, overuse)**
  This variable was included based on prior study inclusion as an exposure variable [8]. Due to very low numbers in the overuse category, this was grouped with atraumatic. Data source: REDCap database
7 **Patient was managed with plaster (plaster, no plaster)**
  A new variable was created from data set variables related to immobilisation of the upper or lower limb (yes/no). This variable was included as it was suggested by clinicians in the MDP as a reason they perceived that people re‐presented, i.e., re‐presenting because of problems with their plaster. Data source: REDCap database
8 **Required Imaging (yes, no)**
  Patients who had imaging (related to their condition) prior to arrival at ED, and those that had imaging at RGH were included in the yes category. Data source: REDCap database
9 **Triage Code (higher priority, lower priority)**
  Initial analysis of individual triage codes demonstrated no relationship between triage code and re‐presentation. There was nil change when categorised as low vs. high priority and therefore reported as such. Data source: EDIS
  – Higher Priority: ATS 2 or 3.
  – Lower Priority: ATS 4 or 5.
10 **Socioeconomic Status**
  This variable was initially recoded in deciles based on the most recent IRSD codes for the relevant postcode [21]. Initial individual analysis demonstrated nil odds of re‐presenting. No IRSD decile had greater odds of re‐presenting. Data source: EDIS
11 **Occupation (Grouped)**
  Initial individual analysis demonstrated only the pensioner group (small numbers) had a significant relationship with re‐presentation. Occupations were grouped into four categories based on logical groupings. There were no heightened odds in any group, thus retained as the following:
  – Other/Unemployed/Pensioner.
  – Student.
  – Sedentary.
  – Standing/Physical/Heavy.

Data source: REDCap database

12 **Pain Severity (EQ‐5D)**
  Pain severity has been included as an exposure variable in prior studies, utilising a variety of outcome tools. From the EQ‐5D, a single item for pain on the five‐point Likert scale was used in this study (no/slight/moderate/severe or extreme pain and discomfort) [11]. Initial coding across 5 categories demonstrated very few numbers in the first and last categories (no or extreme pain). Analysis showed a general linear trend as pain increases with an increase in re‐presentation rate. Categories were collapsed down to three (low, moderate or high pain). The same linear trend remained whilst collapsing categories with low numbers. Data source: REDCap database
13 **Anxiety/Depression Score EQ5D (nil—slight, moderate—extreme)**
  From the EQ‐5D, a single item for anxiety/depression on the five‐point Likert scale was used in this study (not/slightly/moderately/severely or extremely anxious or depressed) [11]. Initial analysis across 5 categories demonstrated nil increased odds for re‐presentation in any category. Categories were collapsed down to two categories (no/slight and moderate/severe/extreme) there was nil change in odds therefore presented as such. Anxiety/depression score was included based on prior study inclusion as an exposure variable. Data source: REDCap database
14 **Health Literacy**
  Data was derived from a single question “How often do you have someone (like a family member, friend, hospital worker or a carer) help you read materials given to you by the hospital?” This question is one of the Single Item Literacy Screener questions recommended for use in ED research [12]. Response categories (never, occasionally, often, always) were collapsed into two categories from response levels: Low = never and occasionally. High = often and occasionally. Data source: REDCap database
15 **Satisfaction (Binary)**
  This variable was included based on prior study inclusion as an exposure variable and is taken from the Short Assessment of Patient Satisfaction (SAPS) [10]. Data source: REDCap database.
  Initial coding across five categories demonstrated very few numbers in the not satisfied category and there was no relationship with increased odd of re‐presentation. When collapsed into two categories there was nil change to relationship and categories with low numbers was collapsed. The single question asked was ‘Overall, I was satisfied with my treatment experience’ = Satisfaction
  – Satisfied = Agree or strongly agree
  – Not satisfied = Neither agree nor disagree, disagree, strongly disagree

## Appendix C

**TABLE C1:** Characteristics of patients that had a scheduled re‐presentation compared with those with an unscheduled re‐presentation.

Variable	Scheduled re‐presentation	Unscheduled re‐presentation	*p* ^b^
	*n* (%)	*n* (%)	
Total	*n* = 65	*n* = 78	
Age^a^	32.4 (16–53)	28.4 (14–51)	0.410
Sex, female	39 (60)	45 (57.7)	0.780
Body area affected			
Upper limb	19 (29.2)	31 (39.7)	0.730
Lower limb	42 (64.6)	45 (57.7)	—
Spinal	4 (6.2)	2 (2.6)	—
Current cigarette smoker, yes	5 (7.7)	7 (9)	0.783
Number of current medications			
Nil	34 (52.3)	44 (56.4)	0.773
1–3	27 (41.5)	31 (39.7)	—
4+	4 (6.2)	3 (3.8)	—
Type of injury, traumatic	58 (89.2)	72 (92.3)	0.526
Plaster immobilisation, yes	1 (1.5)	19 (24.4)	**< 0.001** *****
Received imaging, yes	53 (81.5)	55 (70.5)	0.068
Triage score, ATS, lower acuity [3, 4]	55 (84.6)	66 (84.6)	1.000
Socioeconomic Status, Higher SES	28 (43.1)	30 (38.5)	0.576
Occupation (Grouped)			
Other/unemployed/pensioner	3 (4.6)	11 (14.1)	0.226
Student	29 (44.6)	26 (33.3)	—
Sedentary	8 (12.3)	8 (10.3)	—
Standing/physical/heavy	25 (38.5)	33 (42.3)	—
EQ‐5D pain severity^c^			0.540
Low	6 (15.8)	10 (20.8)	—
Moderate	22 (57.9)	22 (45.8)	—
High	10 (26.3)	16 (33.3)	—
EQ‐5D anxiety/depression^c^			
No or slight	33 (86.8)	38 (79.1)	0.355
Health literacy, high^c^	36 (94.7)	43 (89.5)	0.394
Satisfaction. Satisfied, yes^d^	44 (91.6)	57 (100)	**0.026** *****

*Note:* The bold values indicate a significant level of evidence for association.

^a^ Median, interquartile range.

^b^ *p*‐value for group difference.

^c^ EQ5D, health literacy responses, *n* = 38 scheduled, 48 unscheduled.

^d^ Satisfaction responses, *n* = 48 scheduled, 57 unscheduled.

* **Statistically significant**.
